# Concurrent Dual Biologic Therapy (Infliximab-Risankizumab and Dupilumab-Rituximab) for Complex Treatment-Refractory Dermatologic Disease: A Two-Patient Case Report

**DOI:** 10.1159/cde/abvag004

**Published:** 2026-08-03

**Authors:** Chelsea Butler, Maksym Breslavets

**Affiliations:** a Faculty of Medicine, University of Ottawa, Ottawa, ON, Canada; b Centre for Medical and Surgical Dermatology, Pickering, ON, Canada

**Keywords:** Dual biologic therapy, Paradoxical psoriasis, Atopic dermatitis, Case report

## Abstract

**Introduction:**

The use of targeted biologic therapies has transformed the management of inflammatory dermatoses. However, treatment becomes increasingly complex when patients require simultaneous immunomodulation for coexisting systemic conditions. Evidence guiding the concurrent use of dual biologic therapies remains limited.

**Case Presentations:**

We report two cases illustrating the use of concurrent dual biologic therapy in treatment-refractory dermatologic disease. The first case involves a woman with ulcerative colitis who developed paradoxical psoriasis while receiving infliximab. Discontinuation of infliximab resulted in a gastrointestinal disease flare, necessitating continuation of therapy. Concomitant treatment with risankizumab led to marked improvement in cutaneous disease without adverse effects. The second case describes a woman with severe, treatment-resistant atopic dermatitis receiving maintenance rituximab for indolent non-Hodgkin lymphoma. Dupilumab was initiated following interdisciplinary consultation, resulting in sustained dermatologic remission over long-term follow-up with no treatment-emergent adverse events.

**Conclusion:**

These cases highlight the potential role of carefully selected concurrent dual biologic therapy in managing complex, treatment-refractory dermatologic disease when monotherapy is insufficient and discontinuation of essential systemic treatment is not feasible. Multidisciplinary collaboration and close monitoring are essential when considering such therapeutic strategies.

## Introduction

Targeted biologics and small-molecule inhibitors have revolutionized the treatment of chronic inflammatory skin diseases such as psoriasis and atopic dermatitis (AD). However, therapeutic decision-making becomes increasingly complex when systemic immune modulation is simultaneously required for comorbid conditions, including inflammatory bowel disease and hematologic malignancy [1]. The complexity is significantly increased when two biologic response modifiers (BRMs) are used concurrently, or when an additional BRM is administered to mitigate adverse effects caused by the primary therapeutic BRM in situations where discontinuation of the culprit agent is not feasible due to clinical indications.

Paradoxical psoriasis is a well-recognized adverse effect of tumor necrosis factor-alpha (TNF-α) inhibitors in patients with inflammatory bowel disease, though its immunopathogenesis remains incompletely understood [2]. Similarly, the safety of initiating immunomodulatory therapies such as dupilumab in patients receiving concurrent B-cell-depleting agents, including rituximab, is not well defined [3]. Evidence to inform the use of dual biologic or targeted therapy remains limited [3]. We present two cases that illustrate these therapeutic challenges: one involving infliximab-associated paradoxical psoriasis managed with concomitant risankizumab and another describing dupilumab initiation for severe AD during rituximab maintenance therapy for indolent lymphoma.

## Case Presentation

### Patient 1

A 38-year-old woman with ulcerative colitis (UC) was referred for evaluation of psoriasis that developed while receiving infliximab. Approximately 6–7 months after initiation of infliximab 5 mg/kg intravenously every 8 weeks for UC control, she developed multiple scaly patches and plaques involving the scalp, face, ears, postauricular areas, elbows, forearms, lower back, and extensor aspects of the lower extremities, consistent with clinically diagnosed paradoxical psoriasis. At baseline dermatological assessment in November 2023, the Psoriasis Area and Severity Index (PASI) was 18, and body surface area (BSA) involvement was 11%.

Infliximab was discontinued, but UC flared. Sequential therapies, including ustekinumab and upadacitinib, resulted in partial skin responses but failed to manage UC. Methotrexate was introduced as adjunctive therapy alongside infliximab but was discontinued due to the patient’s intolerance, such as extreme nausea and fatigue. A history of melanoma in situ further limited systemic options, including phototherapy.

Given the complexity of the patient’s presentation, infliximab was continued at 5 mg/kg intravenously every 8 weeks, and risankizumab 150 mg subcutaneously every 12 weeks was initiated as ongoing dual biologic therapy on January 24, 2024. The response was assessed on May 21, 2024, with ongoing regular follow-up by dermatology and gastroenterology. Clinical monitoring included assessment for treatment response, tolerability, infections, and new or concerning cutaneous/systemic symptoms during specialist follow-up. At follow-up in September 2025, residual patches remained only on shins and postauricular regions. PASI improved from 18 to 1.5 (91.7% reduction), and BSA from 11% to 2.3%. The Dermatology Life Quality Index (DLQI) improved from 24 to 1. No adverse events were reported.

### Patient 2

A 45-year-old woman presented in September 2019 with a 4-month history of diffuse, intensely pruritic, treatment-resistant AD. Prior treatments included multiple topical corticosteroids (beclomethasone dipropionate 0.025%, fluocinonide 0.05%, and desoximetasone 0.25%), a topical calcineurin inhibitor (tacrolimus 0.1% ointment), intramuscular triamcinolone (50 mg), and narrowband UV-B phototherapy, all with inadequate response.

Her medical history included scleroderma with Raynaud phenomenon, hypothyroidism, asthma, and low-grade non-Hodgkin lymphoma, for which she received maintenance rituximab 375 mg/m^2^ intravenously every 3 months at the time dupilumab was initiated. The patient later reported completing rituximab therapy in 2023. Dermatologic examination revealed widespread erythematous, lichenified patches with excoriations and marked xerosis (dryness score 3). Baseline disease severity scores included Investigator Global Assessment (IGA) of 4 (severe), Eczema Area and Severity Index (EASI) of 23.4, BSA of 40%, and DLQI of 24.

Given inadequate disease control, dupilumab 300 mg subcutaneously every 2 weeks was initiated in April 2020 after interdisciplinary consultation and continued as ongoing therapy. Rituximab was continued at that time. Initial response was assessed in July 2020, with a 50% reduction in BSA involvement and improvement in Investigator Global Assessment from 4 to 2. Given her lymphoma history, malignancy-related monitoring was performed through oncology follow-up, while dermatology monitored skin disease activity, treatment tolerability, and cutaneous adverse effects. At follow-up in October 2025, she maintained excellent disease control on dupilumab, with EASI 0, BSA 0%, and DLQI 5. No treatment-emergent adverse events were reported during clinical follow-up. In both cases, initial treatment response was assessed within approximately 3 to 4 months after initiation or modification of biologic therapy, followed by regular multidisciplinary follow-up individualized according to disease activity, treatment tolerability, comorbidities, and risk of adverse events.

## Discussion

The management of inflammatory dermatoses in patients with systemic immune-mediated comorbidities presents increasing complexity in the era of targeted immunotherapy. Though biologics and small molecules have dramatically improved care of psoriasis and AD, real-world scenarios sometimes demand simultaneous modulation of distinct immune pathways, an area where evidence is sparse [3].

In patient 1, paradoxical psoriasis emerged during infliximab therapy for UC. The prevailing hypothesis posits that TNF-α blockade leads to unchecked production of interferon-α by plasmacytoid dendritic cells (pDCs), initiating a psoriasiform cascade even in the absence of a classic T cell-driven autoimmune process [4, 5]. Anti-TNF therapy may prolong pDC maturation, sustaining type I interferon expression, which in turn activates myeloid dendritic cells and downstream IL-23/IL-17-driven inflammation [4]. Skin lesions in paradoxical psoriasis have been shown to demonstrate enhanced type I interferon signatures and dermal pDC infiltration compared with classical psoriasis [4]. This innate-immune-driven shift may help explain why paradoxical lesions often fail to follow the memory/relapsing pattern typical of idiopathic psoriasis [6].

From a management standpoint, paradoxical reactions pose a dual dilemma: discontinuing the culprit biologic may ameliorate skin disease but risk a flare of the underlying systemic disease. Conventional strategies include switching classes or adding topical therapy [7]. However, in patients like ours with active colitis requiring continued TNF blockade, combining therapies may be pragmatically necessary. The durable PASI 90 response in our patient on concomitant infliximab and risankizumab suggests that selective, nonredundant dual biologic therapy may be a viable salvage strategy in highly selected scenarios.

In patient 2, the challenge was integrating dupilumab for severe AD into ongoing rituximab maintenance therapy for indolent non-Hodgkin lymphoma. Dupilumab exerts its effect by antagonizing IL-4Rα, thereby inhibiting both IL-4 and IL-13 signaling [8]. Its mechanism is relatively targeted within the type 2 immune axis, which may limit overlap with B-cell-depleting therapies [8]. In one series, patients with prior or concurrent malignancy treated with dupilumab demonstrated good AD control without observed cancer recurrence over-follow-up [9]. In our patient, long-term dupilumab follow-up, including a period of overlap with rituximab maintenance therapy, was associated with durable remission of AD (EASI 0, BSA 0) and no treatment-emergent adverse events reported during clinical follow-up.

Despite these promising observations, caution is warranted. While rare reports have described cutaneous T-cell lymphoma arising during dupilumab therapy, a causal relationship has not been established, and the clinical significance remains controversial, warranting further investigation [10]. Moreover, the long-term immunologic synergy (or potential antagonism) of dual biologic or small-molecule regimens remains poorly characterized [3].

These cases illustrate both the potential and the complexity of combining targeted therapies with distinct immunologic mechanisms. When monotherapy fails to manage coexisting immune conditions, carefully selected dual therapy may be justified. However, until prospective registries or formal trials provide greater insight, such regimens should remain in the domain of carefully selected patients, with rigorous monitoring and shared decision-making.

## Statement of Ethics

Ethical approval is not required for this study in accordance with local or national guidelines. Written informed consent was obtained from the patients for publication of the details of their medical case. Patient consent form was not provided to the journal but is retained by the authors. The CARE Checklist has been completed by the authors for this case report, attached as online supplementary material (for all online suppl. material, see https://doi.org/10.1159/cde/abvag004).

## Conflict of Interest Statement

The authors have no conflicts of interest to declare.

## Funding Sources

This study was not supported by any sponsor or funder.

## Author Contributions

C.B. drafted the manuscript. M.B. provided clinical oversight, acquired the clinical data, and revised/finalized the manuscript. Both authors contributed to the conception of the report, interpreted the data, reviewed previous versions, and approved the final manuscript.

## Data Availability

All data generated or analyzed during this study are included in this article and its online supplementary material. Further inquiries can be directed to the corresponding author.

## References

[1] AAAAI/ACAAI JTF Atopic Dermatitis Guideline Panel; Chu DK, Schneider L, Asiniwasis RN, Boguniewicz M, De Benedetto A, . Atopic dermatitis (eczema) guidelines: 2023 American academy of allergy, asthma and immunology/american college of allergy, asthma and immunology joint task force on practice parameters GRADE– and institute of medicine–based recommendations. Ann Allergy Asthma Immunol. 2024;132(3):274–312. 10.1016/j.anai.2023.11.009 38108679

[2] Bucalo A , Rega F, Zangrilli A, Silvestri V, Valentini V, Scafetta G, . Paradoxical psoriasis induced by Anti-TNFα treatment: evaluation of disease-specific clinical and genetic markers. Int J Mol Sci. 2020;21(21):7873. 10.3390/ijms21217873 PMC7660646 33114187

[3] Lignou AZ , Vassilakis KD, Baraliakos X, Sfikakis PP, Gottenberg JE, Fragoulis GE. Combination targeted therapy with two biologic/targeted synthetic DMARDs in 1200 patients with immune mediated inflammatory diseases. A systematic literature review for current landscape in safety and efficacy. Autoimmun Rev. 2025;24(10):103865. 10.1016/j.autrev.2025.103865 40633795

[4] Chokshi A , Demory Beckler M, Laloo A, Kesselman MM. Paradoxical tumor necrosis factor-alpha (TNF-α) inhibitor-induced psoriasis: a systematic review of pathogenesis, clinical presentation, and treatment. Cureus. 2023;15(8):e42791. 10.7759/cureus.42791 PMC10469896 37664349

[5] Conrad C , Di Domizio J, Mylonas A, Belkhodja C, Demaria O, Navarini AA, . TNF blockade induces a dysregulated type I interferon response without autoimmunity in paradoxical psoriasis. Nat Commun. 2018;9(1):25. 10.1038/s41467-017-02466-4 PMC5750213 29295985

[6] Mylonas A , Conrad C. Psoriasis: classical vs. paradoxical. The Yin-Yang of TNF and type I interferon. Front Immunol. 2018;9:2746. 10.3389/fimmu.2018.02746 PMC6283263 30555460

[7] Camela E , Potestio L, Fabbrocini G, Megna M. Paradoxical reactions to biologicals for psoriasis. Expert Opin Biol Ther. 2022;22(12):1435–7. 10.1080/14712598.2022.2153593 36445319

[8] Harb H , Chatila TA. Mechanisms of dupilumab. Clin Exp Allergy. 2020;50(1):5–14. 10.1111/cea.13491 PMC6930967 31505066

[9] Tanczosova M , Hugo J, Gkalpakiotis S. Treatment of severe atopic dermatitis with dupilumab in patients with advanced cancer. J Clin Med. 2023;12(3):1191. 10.3390/jcm12031191 PMC9918040 36769838

[10] Hasan I , Parsons L, Duran S, Zinn Z. Dupilumab therapy for atopic dermatitis is associated with increased risk of cutaneous T cell lymphoma: a retrospective cohort study. J Am Acad Dermatol. 2024;91(2):255–8. 10.1016/j.jaad.2024.03.039 38588818

